# Effect of accreditation on the quality of chronic disease management: a comparative observational study

**DOI:** 10.1186/s12875-014-0179-4

**Published:** 2014-11-04

**Authors:** Arna L van Doorn – Klomberg, Jozé CC Braspenning, René J Wolters, Margriet Bouma, Michel Wensing

**Affiliations:** Radboud University Medical Center, Scientific Institute for Quality of Healthcare, PO Box 9101, 6500 HB Nijmegen, The Netherlands; Dutch College of General Practitioners (NHG), Utrecht, The Netherlands

**Keywords:** Accreditation, Chronic disease, Primary health care, Quality improvement

## Abstract

**Background:**

Practice accreditation is widely used to assess and improve quality of healthcare providers. Little is known about its effectiveness, particularly in primary care. In this study we examined the effect of accreditation on quality of care regarding diabetes, chronic obstructive pulmonary disease (COPD) and cardiovascular disease (CVD).

**Methods:**

A comparative observational study with two cohorts was performed. We included 138 Dutch family practices that participated in the national accreditation program for primary care. A first cohort of 69 practices was measured at start and completion of a 3-year accreditation program. A second cohort of 69 practices was included and measured simultaneously with the final measurement of the first cohort. In separate multilevel regression analyses, we compared both within-group changes in the first cohort and between-groups differences at follow-up (first cohort) and start (second cohort). Outcome measures consisted of 24 systematically developed indicators of quality of care in targeted chronic diseases.

**Results:**

In the within-group comparison, we found improvements on 6 indicators related to diabetes (feet examination, cholesterol measurement, lipid lowering medication prescription) and COPD (spirometry performance, stop smoking advice). In the between-groups comparison we found that first cohort practices performed better on 4 indicators related to diabetes (cholesterol outcome) and CVD (blood pressure outcome, smoke status registration, glucose measurement).

**Conclusions:**

Improvements of the quality of primary care for patients with chronic diseases were found, but few could be attributed to the accreditation program. Further development of accreditation is needed to enhance its effectiveness on chronic disease management.

## Background

A range of strategies has been developed to assess and improve healthcare for patients with chronic diseases [1]. A widely used strategy is accreditation of healthcare providers [2,3]. Accreditation affects the institution or practice and is offered more or less voluntary, while certification focuses on a specific norm that should be reached by individuals or particular services [4,5]. A key element of accreditation is audit and feedback. A Cochrane review suggested that an audit and feedback system has a small positive effect on quality of care overall [6], but the added value of accreditation was not considered. Greenfield et al. [7] suggested in their systematic review that accreditation can promote change, for example through the opportunity to reflect on organizational performance, and that it has an effect on professional development. However, they reported inconsistent findings regarding quantifiable effects of accreditation on measures of clinical processes and outcomes. Few rigorous evaluations of effectiveness of accreditation are available, particularly in primary care [8-10]. O’Beirne et al. [10] performed a review of peer-reviewed and grey literature regarding accreditation in primary care. They found indications that accreditation may improve both organization and outcomes of care, but conclude that more research is needed. Szecsenyi et al. [11] examined an accreditation program that focuses on practice management; they found improvements on several quality and safety measures regarding complaint management, analysis of critical incidents and quality development. Given the shortage of controlled evaluations, more research on the effects of well-defined accreditation programs is required.

In this study we assessed an accreditation program for primary care practices [12,13], set up by the Dutch college of General Practitioners in 2005. This program strongly focuses on the educational value of audit and feedback and participation is voluntary. However, part of the program does require meeting specific norms, for example on hygiene, first aid equipment and accessibility. The program addresses clinical care as well as practice management. The clinical care section consists mainly of three chronic care conditions: diabetes mellitus, chronic obstructive pulmonary disease (COPD) and cardiovascular disease (CVD).

In the same period, health insurers developed bundled payment schemes, with an initial focus on diabetes (approximate start in 2008), and in later years also on COPD (since 2009) and CVD (since 2011) [14,15]. Bundled payment is suggested as a method to control costs while providing more patient centered, higher quality care [16]. An international review of different types of bundled payment [17] suggests that there is weak but consistent evidence for a positive effect of bundled payment on healthcare costs, with no tangible impact on quality of care. Studies on the effect of bundled payment in the Netherlands show that it improved organization and management of care [18,19] and potentially also the outcomes of diabetes care [20]. Nevertheless, a Cochrane review concluded that there is insufficient evidence to draw firm conclusions on the effectiveness of different these types of reimbursement schemes [21].

This study is focused on effects of the Dutch practice accreditation program in the context of a healthcare system, in which provider reimbursement of chronic illness care was improved simultaniously. In the Netherlands, approximately 80% of diabetes patients has the family physician (FP) as the principal caregiver. This percentage is slightly lower for COPD and CVD patients (50-65%). Generally, patients with the FP as main care provider tend to be stable, although not necessarily well controlled. The study aimed to determine whether participation in the accreditation program led to improvements in quality of care regarding diabetes, chronic obstructive pulmonary disease (COPD) and cardiovascular disease (CVD). We also aimed to assess whether the improved performance was different from the performance during pre-assessment of a second group of participants.

## Methods

### Study design

We performed a comparative observational study with two cohorts of primary care practices. A first cohort of practices was followed throughout the 3-year program with extended measurements at start and after three years. A second cohort was measured at their start of the accreditation program, during the same period of the post-assessment in the first cohort. Data collected at the start of the practice accreditation process portrayed practice performance prior to the accreditation process: data were collected retrospectively over a one year period prior to the start. We evaluated the effect of practice accreditation in a design with two separate comparisons. In the first comparison we assessed improvements over time within the first cohort. In the second comparison we assessed differences between the first and second cohort by comparing the follow-up measurement of the first cohort with the start measurement of the second cohort. Since both of these groups collected data in the same period, this comparison could offer additional information on effects of accreditation (3 years experience versus start of accreditation). The ethics committee of the Radboud university medical center provided a waiver for the study.

### Study population

Practices included in the study were Dutch primary care practices that participated in the accreditation program of the Dutch College of General Practitioners between 2006 and 2011. We included a group of 69 practices in the first cohort that joined the accreditation program before 2009 and had collected follow-up data. Start measurements took place between 2006 and 2008; follow-up measurements of this cohort took place between 2009 and 2011. The average time period between data collection in the first cohort was four years and two months, with a minimal interval of 3 years and 2 months.

**Figure 1:**
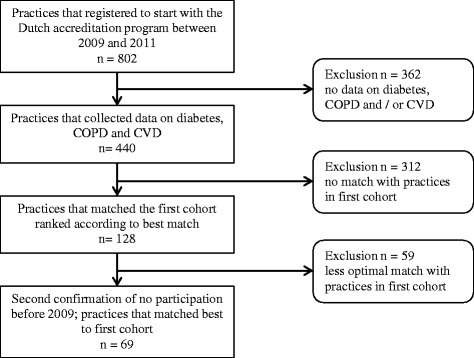
**Selection of practices in the second cohort.**

The second cohort consisted of primary care practices that had their start measurement between 2009 and 2011. We used a matched design to select an equally large sample of 69 practices (see Figure 1). Matching was based on availability of data, practice location (degree of urbanization), visitation date, practice type, and practice size.

### Practice accreditation

Practices have been invited to participate voluntarily in the Dutch practice accreditation program since 2005. The preparatory phase of the program consisted of data collection regarding practice management and patient care. The measurement instruments used were previously validated questionnaires such as the ‘VIP’ , a visitation instrument for practice organization [22] and the ‘Europep’ [23] for patient experiences with care. The clinical performance indicators were derived from the national guidelines valid at the time of measurement [24]. These indicators were measured with the use of patient information that was extracted from electronic medical records; the family physician (FP) or nurse extracted the information either automatically or manually with a standardized extraction form. After data submission via an online system, the practice received a report that included information on their own performance and the performance of other practices as a benchmark. This information could be used to identify which areas needed improvement. The FPs wrote improvement plans with a plan-do-study-act cycle. The first audit was carried out after the approval of these plans to confirm adequate participation and to grant accreditation. After this audit a three-year accreditation cycle started. At the end of each year the practice staff evaluated whether the objectives of improvement programs were met and wrote new improvement plans for the following year. The prolongation of accreditation depended on this process; accreditation was not based on the actual quality of care itself, but rather on the quality of the improvement initiatives according to a structured program.

### Performance indicators

The primary care practice maintains comprehensive patient records, which includes information on all contacts and procedures in primary care, hospital, and several other care providers. For this study, we excluded all patients of which the FP was not the main care provider. All information used in this study was collected as part of the accreditation program. The prevalence of diabetes and COPD was defined as the number of patients with the condition divided by the total number of patients in the practice. Indicators of both process of care and intermediate outcomes were included regarding patients with diabetes, COPD and CVD (Table 1). The data that were collected before 2009 on CVD related to a broader range of patients; patients with high risk for CVD were also included. Since this made it impossible to compare data over time we only included data after 2009, that relate to patients with known CVD. During our research period several target levels as described in the guidelines shifted for diabetes (relating to indicators 2 and 3) and for CVD (indicator 2). We calculated the percentage of patients with a level below target level according to the target level that was valid at the time of measurement. Data on practice characteristics were collected through the use of online questionnaires which were also part of the practice accreditation program.

**Table 1:** **Description of performance scores**

**Topic**	**No.**	**Description**
**Diabetes**		
*Outcome*	1	Percentage of patients with an HbA1c level within target level *(<53 mmol/mol)*
	2	Percentage of patients with a blood pressure within target level *(2006-2008: <150/80 mm Hg, 2009-2011: systolic <140 mm Hg)*
	3	Percentage of patients with a total cholesterol level within target level *(2006-2008: <5.0 mmol/l, 2009-2011: <4.5 mmol/l)*
*Process*	4	Calculated mean process score based on the following 6 performance measures:
	4a	Percentage of patients with at least one HbA1c measurement in the past 12 months
	4b	Percentage of patients with at least one blood pressure measurement in the past 12 months
	4c	Percentage of patients with at least one cholesterol measurement in the past 12 months
	4d	Percentage of patients with a creatinine clearance calculation in the past 12 months
	4e	Percentage of patients with a retinal examination in the past 24 months
	4f	Percentage of patients with a feet examination in the past 12 months
	5	Percentage of patients with a current prescription of lipid lowering medication
**Chronic obstructive pulmonary disease (COPD)**		
*Outcome*	1	Percentage of patients that smoke
*Process*	2	Calculated mean process score based on the following 2 performance measures:
	2a	Percentage of patients with a known smoke status
	2b	Percentage of patients with a spirometry in the past 12 months
	3	Percentage of patients that smoke with a stop smoking advice
**Cardiovascular disease (CVD)**		
*Outcome*	1	Percentage of patients that smoke
	2	Percentage of patients with a blood pressure level within target level *(2006-2008: <160/90 mm Hg, 2009-2011: systolic <140 mm Hg)*
*Process*	3	Calculated mean process score based on the following 2 performance measures:
	3a	Percentage of patients with a known smoke status
	3b	Percentage of patients with at least one blood pressure measurement in the past 12 months
	4	Percentage of patients that smoke with a stop smoking advice
	5	Percentage of patients with a glucose measurement in the past 12 months
	6	Percentage of patients with a current prescription of anticoagulants

### Analyses

Descriptive statistics were used to describe the practice population and gain insight in the performance scores. For each of the three conditions we calculated a mean score for several process indicators. Some indicators could not be included in the mean scores due to low internal consistency with other indicators or use of subsamples: diabetes indicator 5, COPD indicator 3 and CVD indicators 4, 5 and 6 (Table 1).

In order to examine whether chronic care management improved after the three year cycle of the accreditation program, we compared the start and follow-up scores of the first cohort in a within-subject design. We performed separate multilevel regression analyses for each indicator. We accounted for repeated measures per practice and included predictors reflecting practice size, practice type and practice location.

Practices in the first cohort were compared to practices in the second cohort on follow-up and baseline scores respectively, in a separate regression analysis. The comparison was corrected for the match criteria practice size, practice type and practice location in each model. All analyses were performed with SPSS version 20 [25].

## Results

### Practice characteristics

Table 2 shows the percentages and means of different practice characteristics in each group. There was a slight underrepresentation of practices that were part of a health center in the second cohort compared to the first cohort: 8 (12%) versus 11 (16%) practices. They were slightly more often located in an urban area (25 (36%) versus 23 (33%) practices). The mean number of patients in the practice increased in time; mean practice size was similar in the two cohorts.

**Table 2:** **Practice characteristics**

	**Practices in first cohort**				**Practices in second cohort**	
**Practice characteristic**	**2006-2008**		**2009-2011**		**2009-2011**	
	**N**	**%**	**N**	**%**	**N**	**%**
Practice type						
Single handed	17	25%	18	26%	19	27%
Health center	10	14%	11	16%	8	12%
Other	42	61%	40	58%	42	61%
Practice location^1^						
Urban	23	33%	23	33%	25	36%
Semi-urban	34	49%	34	49%	33	48%
Rural	12	18%	12	18%	11	16%
Average number of patients per practice	4629		4808		4830	

^1^A region was defined as urban when the number of addresses per km^2^ exceeded 1500. Semi-urban regions had between 500 and 1500 addresses per km^2^, rural regions had less than 500 addresses per km^2^.

### Within-group comparison

The first two columns in Table 3 report on scores at start and follow-up of practices in the first cohort. The recorded prevalence of diabetes increased from 40 to 50 per 1000 patients. The recorded prevalence of COPD also increased, from 17 to 22 per 1000 patient. The prevalence of CVD/CVRM was not measured.

**Table 3:** **Within-practice comparison of first and second measurement of practices in first cohort and between practice comparison of first and second cohort in 2009-2011**

**Indicators** ^**1**^	**1** ^**st**^ **cohort**		**Within-group comparison of 1** ^**st**^ **cohort** ^**2**^		**2** ^**nd**^ **cohort**	**Between-groups comparison 1** ^**st**^ **and 2** ^**nd**^ **cohort 2009-2011** ^**3**^	
	**2006-2008**	**2009-2011**			**2009-2011**		
	**Mean (SD)**	**Mean (SD)**	**Estimate (95% conf. int.)**	**p**	**Mean (SD)**	**Estimate (95% conf. int.)**	**p**
**Diabetes**							
Prevalence	0.040 (0.014)	0.050 (0.013)	0.010 (0.006 - 0.015)	0.000	0.051 (0.018)	-0.001 (-0.006 - 0.005)	0.79
*Outcome measures*							
HbA1c < target	60.8 (15.9)	63.6 (11.4)	2.6 (-2.6 - 7.9)	0.32	63.6 (11.0)	-0.02 (-4.0 - 3.9)	0.99
Blood pressure < target^4^	56.6 (16.7)	52.1 (17.0)	-5.4 (-11.4 - 0.7)	0.08	50.8 (16.7)	0.9 (-4.9 - 6.7)	0.76
Total cholesterol < target^4^	50.4 (15.0)	45.2 (20.1)	-5.4 (-12.4 - 1.5)	0.12	36.3 (20.7)	8.8 (1.5 - 16.1)	0.02
*Process measures*							
Mean process score	79.2 (10.0)	81.6 (12.7)	2.3 (-1.5 - 6.1)	0.23	79.5 (12.7)	1.8 (-2.5 - 6.2)	0.41
HbA1c measurement	90.4 (10.2)	92.5 (7.3)	2.1 (-1.1 - 5.4)	0.20	92.4 (6.7)	-0.03 (-2.5 - 2.4)	0.98
BP^5^ measurement	93.7 (7.0)	91.9 (13.3)	-1.7 (-5.0 - 1.7)	0.32	92.4 (8.9)	-0.7 (-4.7 - 3.3)	0.74
Cholesterol measurement	81.3 (14.4)	86.1 (12.4)	4.8 (0.2 - 9.4)	0.04	86.6 (9.4)	-0.6 (-4.4 - 3.2)	0.74
Creatinine clearance	82.1 (13.1)	85.3 (17.0)	3.4 (-1.2 - 7.9)	0.14	83.6 (17.3)	1.2 (-4.8 - 7.2)	0.69
Retinal examination	70.6 (16.4)	66.4 (24.4)	-4.5 (-12.0 - 3.1)	0.24	62.3 (26.6)	3.9 (-4.9 - 12.7)	0.39
Feet examination	57.3 (22.7)	67.1 (26.4)	9.7(0.9 - 18.5)	0.03	59.7 (27.1)	7.2 (-2.1 - 16.5)	0.13
Prescription of lipid lowering medication	60.5 (16.4)	69.9 (22.6)	9.5 (2.5 - 16.4)	0.009	68.4 (13.1)	1.1 (-5.1 - 7.4)	0.72
**Chronic obstructive pulmonary disease (COPD)**							
Prevalence	0.017 (0.009)	0.022 (0.008)	0.005 (0.003 - 0.008)	0.000	0.023 (0.010)	-0.001 (-0.004 - 0.003)	0.65
*Outcome measure*							
Patients that smoke	36.6 (22.9)	31.8 (16.1)	-4.9 (-11.5 - 1.8)	0.15	32.2 (20.7)	-0.4 (-6.9 - 6.2)	0.92
*Process measures*							
Mean process score	58.5 (17.8)	67.2 (22.1)	8.3 (1.8 - 14.8)	0.01	61.6 (24.9)	5.5 (-2.8 - 13.8)	0.19
Smoke status known	76.0 (21.4)	75.4 (23.2)	-1.3 (-8.1 - 5.6)	0.71	69.4 (27.0)	6.1 (-2.8 - 15.0)	0.18
Spirometry	41.2 (23.5)	58.8 (28.7)	17.0 (8.0 - 26.0)	0.000	53.8 (28.5)	4.7 (-5.5 - 14.8)	0.37
Stop smoking advice	47.0 (33.9)	69.8 (30.8)	21.8 (8.7 - 34.9)	0.002	65.2 (32.6)	5.1 (-10.1 - 20.2)	0.51
**Cardiovascular disease (CVD)** ^**6**^							
*Outcome measures*							
Patients that smoke	-	12.6 (8.5)	-	-	10.5 (7.8)	1.9 (-1.1 - 4.9)	0.20
Blood pressure < target^4^	-	41.8 (15.5)	-	-	35.7 (14.7)	6.3 (0.6 - 11.9)	0.03
*Process measures*							
Mean process score	-	61.3 (20.3)	-	-	54.5 (21.6)	6.5 (-1.2 - 14.1)	0.10
Smoke status known	-	51.7 (26.6)	-	-	39.8 (25.5)	11.3 (1.9 - 20.8)	0.02
BP^5^ measurement	-	70.8 (17.9)	-	-	69.3 (21.3)	1.6 (-5.6 - 8.9)	0.66
Stop smoking advice	-	66.7 (34.2)	-	-	51.1 (34.0)	13.2 (-4.6 - 30.9)	0.14
Glucose measurement	-	77.5 (17.6)	-	-	64.3 (24.3)	12.0 (1.9 - 22.0)	0.02
Prescription of anticoagulants	-	78.8 (15.0)	-	-	76.5 (20.4)	1.6 (-5.1 - 8.3)	0.64

^1^Except for prevalence values, all values in this table report the percentage of patients treated according to the guidelines/for which the indicator was met (deviations, estimates and 95% confidence intervals are also reported in number of percentage points). ^2^Results are based on multilevel regression analyses, accounting for repeated measures per practice and correcting for practice size, practice type and practice location. ^3^Results are based on regression analyses, correcting for practice size, practice type and practice location. ^4^In prevailing guidelines, target levels on blood pressure and cholesterol were tightened over time. This may partly account for a decrease in these scores regarding diabetes. ^5^BP = blood pressure. ^6^The inclusion criteria for patients with risk for cardiovascular disease changed towards the inclusion of patients with known cardiovascular disease only, which made a within-group comparison of the first cohort not justifiable.

Table 3 also reports results from the multilevel regression analyses. Regarding diabetes we found that practices had improved on three performance indicators. Practice scores had higher percentages of patients with a recorded cholesterol measurement (p 0.04), feet examination (p 0.03) and prescription of lipid lowering medication (p 0.009). The percentage of patients with a blood pressure below target level was marginally lower at follow up (p 0.08). Practices in the first cohort had improved on three COPD process indicators. There was an increase in the percentage of patients with a recorded spirometry measurement (p 0.000), which was the sole contributor to the effect on the mean process score (p 0.01). We also found a large improvement in the percentage of patients who smoke that received an advice to stop smoking (p 0.002).

### Between-groups comparison

The figures for the between-groups comparison are reported in the columns on the right side of Table 3. No differences were found in the recorded prevalence of diabetes and COPD.

Diabetes patients in the second cohort less often had a total cholesterol value within target level compared to the patients in the first cohort (p 0.02). No other differences were found between the two cohorts regarding diabetes. We did not find any differences on COPD performance indicators. Practices in the first cohort provided higher quality of care than the second cohort on three CVD performance indicators. A higher percentage of patients had a blood pressure within target level (p 0.03). Furthermore, the number of patients with recorded smoke status (p 0.02) and glucose values (p 0.02) had increased.

## Discussion

### Summary

Improvements were found regarding cholesterol measurement, feet examination and the prescription of lipid lowering medication (diabetes), spirometry performance and the provision of a stop smoking advice (COPD). As the follow-up measurement scores on these performance measures were similar to the baseline scores in the second cohort, it remains uncertain whether the effect can be attributed to the accreditation program. Compared to the second cohort of newly starting practices, the practices in the first cohort performed better regarding achievement of a target cholesterol level (diabetes) and blood pressure level (CVD) and the registration of smoke status and measurement of glucose levels (CVD). These differences could provide evidence for the added value of the accreditation program.

### Explanation of findings

Our results are consistent with previous studies that show that there might be an effect of accreditation on quality of care processes and outcomes, although effects are not always consistent [6,7,10]. Furthermore, we mainly found improvements over time on those measures that had a lower score at baseline, which is consistent with findings from a systematic review on quality improvement strategies [26]. Several scores, especially regarding diabetes, were already quite high, which might be a reflection of the general amount of attention towards diabetes care improvement, but it can also be related to the relatively large numbers of practices that provide vocational training in our study population, which is associated with better quality of care [27]. In the Netherlands, approximately 30% of all practices have at least one family physician that provides training. In our population the percentage of practices with training was higher in both cohorts: 61% (42 of 69 practices) and 70% (48 of 69) in the first and second cohort respectively.

In the Dutch accreditation program, the practice is accredited if it is actively involved in quality improvement through use of improvement plans and can achieve improvements over time. An advantage of this approach is that the program is more attractive for health professionals; they take ownership of the improvement plans that are tailored to the individual practices. Like other accreditation programs, it uses audit and feedback as a central mechanism, embedded in a continuous quality improvement process that aims to enhance a culture that fosters quality and safety of healthcare [4,28]. The fact that we did not find stronger evidence for an added value of the accreditation program may be partially explained by other developments in the primary care field during our research period. Starting in 2007, bundled payment was implemented broadly for diabetes and to a more limited extent for COPD; implementation of bundled payment for CVD just started at the end of our research period (2011). Although a large part of the practices may currently participate in bundled payment schemes [15], during our study period, most schemes were only just set up. Bundled payment offers audit and feedback and funds for support/education etcetera, but no other interventions to achieve quality improvement. The Dutch accreditation program is one example of an intervention that can complement further implementation through the development of improvement plans and continuous support. There are clear similarities between participation in bundled payment and the accreditation process as an audit and feedback system. Both require data collection and offer feedback; measures used on diabetes, COPD and CVD are almost identical. In fact, practices that participate in both initiatives often collect data only once and use the data for both purposes. This might explain why little added value of the accreditation program was detected for diabetes and COPD, two conditions for which many practices also participate in a bundled payment initiative. It could also account for the effect of accreditation on CVD, since implementation of bundled payment for this condition only just started during our research period.

### Strenghts and limitations

Our study design implied a risk of confounding, because of the absence of random allocation. The second cohort was selected out of a possible 802 practices of all 4917 Dutch practices (16.3%) [29]. At the end of our study period, more than half of all Dutch general practitioners (over 4600) worked in a practice that took part in the accreditation program and more than 40% of all practices were accredited. It is likely that a substantial part of these practices also participated in bundled payment for diabetes and COPD; the lack of exact information on this limited this study. In our models, we accounted for practice type, size and location. However, there may have been an underrepresentation of practices with a less than average interest in quality improvement, especially in the first cohort, since participation in the program was voluntary. Both the argument of confounding and of the representation of the study population reflect on the possibility that the effects could be more present in the whole population of general practices.

The increase in diabetes and COPD prevalence between 2006 and 2011 may indicate that practices have improved their registration. On the other hand it can also be a result of efforts to screen the population for undiagnosed patients. Process indicator scores might decrease when registration is better, since there is an association between performing a measurement and registering it. This can cause a bias, which may differ when registration improves. Outcome scores might increase after active screening of the population, due to the inclusion of patients with a mild form of diabetes or COPD.

## Conclusions

General practices improved the quality of provided healthcare for patients with the targeted chronic diseases. Nevertheless, only a few improvements could be attributed to the accreditation program, which may be caused by the fact that other programs address quality of chronic illness care at the same time. Expectations of the effects of accreditation were high among participants and stakeholders, but the results of this evaluation do not support these fully. Continuous monitoring may have been beneficial for practices in order to maintain a high level of quality in some areas and improve further in others. After the study was completed, adaptations to the program were made, which reduced the burden of work and may improve the effectiveness. For instance, these could stimulate practices to not focus solely on the measured items [30].

## Abbreviations

COPD: Chronic obstructive pulmonary disease
CVD: Cardiovascular disease
FP: Family physician
NHG: Dutch abbreviation for Dutch College of General Practitioners
